# Functional analysis of CYP71AV1 reveals the evolutionary landscape of artemisinin biosynthesis

**DOI:** 10.3389/fpls.2024.1361959

**Published:** 2024-03-21

**Authors:** Fang-Yan Chen, Qiu-Yan Mu, Bing-Yi Xu, Yu-Chen Lei, Hui-Ying Liu, Xin Fang

**Affiliations:** ^1^ Shandong Laboratory of Yantai Drug Discovery, Bohai Rim Advanced Research Institute for Drug Discovery, Yantai, Shandong, China; ^2^ State Key Laboratory of Drug Research, Shanghai Institute of Materia Medica, Chinese Academy of Sciences, Shanghai, China; ^3^ State Key Laboratory of Phytochemistry and Plant Resources in West China, Kunming Institute of Botany, Chinese Academy of Sciences, Kunming, China; ^4^ School of Life Sciences, Yunnan University, Kunming, China; ^5^ School of Chemical Science and Technology, Yunnan University, Kunming, China

**Keywords:** artemisinin, Asteraceae sesquiterpene lactone, cytochrome P450 monooxygenases, amorpha-4, 11-diene oxidase, germacrene A oxidase, molecular evolution, loss-of-function event

## Abstract

Artemisinin biosynthesis, unique to *Artemisia annua*, is suggested to have evolved from the ancestral costunolide biosynthetic pathway commonly found in the Asteraceae family. However, the evolutionary landscape of this process is not fully understood. The first oxidase in artemisinin biosynthesis, CYP71AV1, also known as amorpha-4,11-diene oxidase (AMO), has specialized from ancestral germacrene A oxidases (GAOs). Unlike GAO, which exhibits catalytic promiscuity toward amorpha-4,11-diene, the natural substrate of AMO, AMO has lost its ancestral activity on germacrene A. Previous studies have suggested that the loss of the GAO copy in *A. annua* is responsible for the abolishment of the costunolide pathway. In the genome of *A. annua*, there are two copies of AMO, each of which has been reported to be responsible for the different product profiles of high- and low-artemisinin production chemotypes. Through analysis of their tissue-specific expression and comparison of their sequences with those of other GAOs, it was discovered that one copy of AMO (AMOHAP) exhibits a different transcript compared to the reported artemisinin biosynthetic genes and shows more sequence similarity to other GAOs in the catalytic regions. Furthermore, in a subsequent *in vitro* enzymatic assay, the recombinant protein of AMOHAP unequivocally demonstrated GAO activity. This result clearly indicates that AMOHAP is a GAO rather than an AMO and that its promiscuous activity on amorpha-4,11-diene has led to its misidentification as an AMO in previous studies. In addition, the divergent expression pattern of AMOHAP compared to that of the upstream germacrene A synthase may have contributed to the abolishment of costunolide biosynthesis in *A. annua*. Our findings reveal a complex evolutionary landscape in which the emergence of a new metabolic pathway replaces an ancestral one.

## 1 Introduction

Plant secondary metabolites, also known as plant specialized metabolites, are often specific to certain lineages, making their biosynthesis pathways highly evolvable compared to those of primary metabolites. Nevertheless, our understanding of the evolution of these biochemicals is still limited (Weng et al., 2012). One such secondary metabolite is artemisinin, an endoperoxide sesquiterpene lactone that is effective against chloroquine-resistant strains of *Plasmodium falciparum*. Artemisinin is exclusively produced by *Artemisia annua* L. (Asteraceae) (Chen and Xu, 2016; Ikram and Simonsen, 2017; Kung et al., 2017; Wang et al., 2019). On the other hand, a group of structurally diverse sesquiterpene lactones, including guaianolides, eudesmanolides, and germacranolides, which feature an α-methylene γ-lactone moiety, are produced by other plants in the Asteraceae family. These sesquiterpene lactones are collectively known as Asteraceae sesquiterpene lactone (ASTL) (de Kraker et al., 2002). It has been suggested that the biosynthesis of artemisinin is derived from the ASTL pathway (Nguyen et al., 2010, 2019), providing an opportunity to study the dynamics of metabolic pathway evolution.

**Figure 1:**
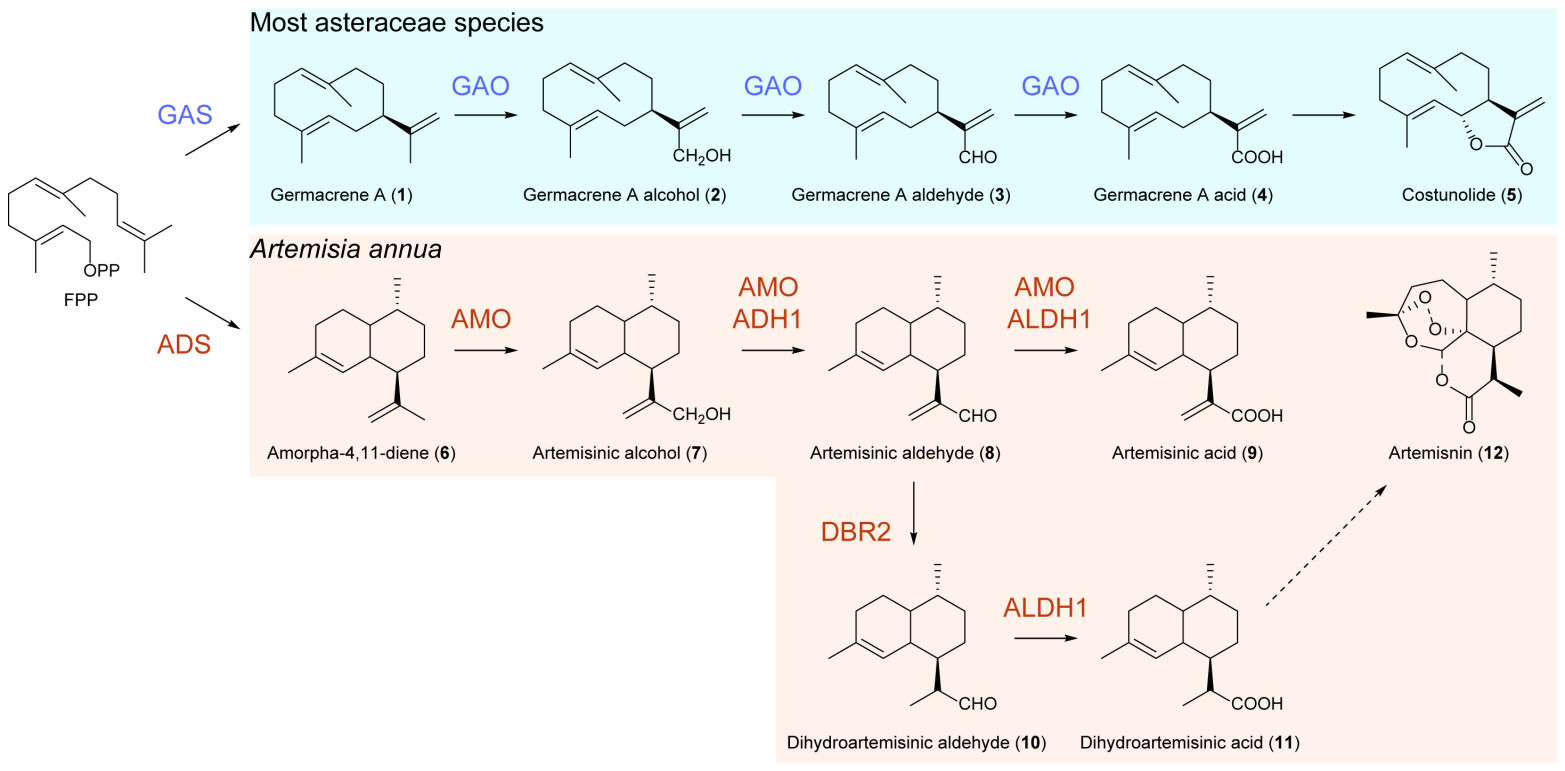
Biosynthetic pathways of artemisinin and costunolide.

The biosynthetic pathways of artemisinin begin with amorpha-4,11-diene synthase (ADS), which converts farnesyl diphosphate (FPP) into the amorphadiene skeleton (Bouwmeester et al., 1999; Chang et al., 2000; Mercke et al., 2000; Huang and Fang, 2021). Subsequently, a cytochrome P450-dependent monooxygenase known as CYP71AV1 performs a three-step oxidation of amorpha-4,11-diene, resulting in the generation of minor products of artemisinic alcohol and artemisinic aldehyde and a major product of artemisinic acid in yeast (Ro et al., 2006). However, in an *in vitro* enzymatic assay using microsomes containing CYP71AV1 and the substrate amorpha-4,11-diene, only a major product of artemisinic alcohol was found, as well as a minor product of artemisinic aldehyde sometimes (Teoh et al., 2006). This enzyme is referred to as amorpha-4,11-diene oxidase (AMO) (**Figure 1**). Notably, there are two additional enzymes also involved in these oxidations in *A. annua*, namely, an alcohol dehydrogenase (ADH1) that catalyzes the conversion of artemisinic alcohol to artemisinic aldehyde and an aldehyde dehydrogenase (ALDH1) that transforms artemisinic aldehyde to artemisinic acid (Liao et al., 2022). Since the Δ11(13) double bond in the above precursors is replaced by a methyl group in artemisinin, artemisinic aldehyde Δ11(13) reductase (DBR2) catalyzes the reduction of artemisinic aldehyde to dihydroartemisinic aldehyde, which is subsequently oxidized to dihydroartemisinic acid by ALDH1 (Liao et al., 2022). Finally, a light-dependent non-enzymatic spontaneous autoxidation converts dihydroartemisinic acid to artemisinin (Czechowski et al., 2018).

The biosynthesis of ASTL shares a conserved core pathway involving germacrene A synthase (GAS) and germacrene A oxidase (GAO), both of which collectively transform FPP to germacrene A acid, a key intermediate of ASTL biosynthesis. GAO catalyzes a similar three-step oxidation reaction to convert germacrene A to a single product, germacrene A acid, in yeast (**Figure 1**) (Bennett et al., 2002; Nguyen et al., 2010; Cankar et al., 2011; Ramirez et al., 2013; Eljounaidi et al., 2014; Nguyen et al., 2019). Interestingly, the GAOs from other plants of Asteraceae exhibit high homology with AMO, and their corresponding proteins can utilize amorpha-4,11-diene as a substrate to produce artemisinic acid as a single product in yeast. Conversely, the AMO protein is inactive toward germacrene A (Nguyen et al., 2010; Cankar et al., 2011; Nguyen et al., 2019). Based on the fact that the activity of ADS is exclusively found in *A. annua*, a prevalent theory postulates that AMO evolved from GAO, gradually acquiring specificity for amorpha-4,11-diene in *A. annua* (Nguyen et al., 2019). This theory provides an explanation for the promiscuous activity of GAO toward amorpha-4,11-diene and the loss of ancestral activity on germacrene A during the specialization of AMO. Consequently, the absence of a GAO copy in the genome of *A. annua* resulted in the elimination of germacrene A acid and ASTL production, despite the presence of germacrene A and functional GAS expression in glandular secreting trichomes (GSTs) (Bertea et al., 2006) where artemisinin is synthesized and accumulated.

In the *A. annua* genome, two copies of *CYP71AV1* can be found. One is a long version with a seven-amino acid extension at the N-terminus of the protein sequences, which was initially isolated and highly expressed in a low-artemisinin production (LAP) chemotype. This long version is referred to as *AMOLAP* (GenBank: PWA40082.1). The other copy is a shorter version that was primarily cloned and dominantly expressed in a high-artemisinin production (HAP) chemotype, which is named *AMOHAP* (GenBank: PWA47004.1) (Ting et al., 2013; Shen et al., 2018; Liao et al., 2022). When expressed in *Nicotiana benthamiana*, *AMOLAP* demonstrated higher enzymatic activity compared to *AMOHAP* in amorpha-4,11-diene (Ting et al., 2013). This suggests that the differential high expression of these two variants in LAP and HAP strains contributes to the different metabolomic profiles of the two chemotypes (Czechowski et al., 2018). However, in another high-artemisinin content chemotype (HAN1), the transcript level of *AMOLAP* was higher than that of *AMOHAP*, which contradicts the previous findings (Liao et al., 2022). It is important to note that, in the above experiment, the AMOLAP form of GAO was selected to examine its cross-activity on germacrene A (Nguyen et al., 2010). However, the activity of AMOHAP on germacrene A was not evaluated. Therefore, the real function of AMOHAP remains to be elucidated.

In this study, we demonstrate that AMOHAP possesses the ability to catalyze the conversion of germacrene A into germacrene A alcohol, providing evidence that AMOHAP indeed functions as a GAO rather than an AMO. Furthermore, the promoter regions of *AMOLAP* and *AMOHAP* exhibit distinct *cis*-elements, indicating divergent transcriptional regulation between these two genes. Therefore, these findings strongly imply that the inability of *A. annua* to produce ASTL is primarily attributed to the altered transcriptional regulation of GAO rather than the loss of GAO function.

## 2 Materials and methods

### 2.1 Chemicals

Farnesyl pyrophosphate ammonium salt, hexane, and NADPH were purchased from Sigma-Aldrich (St. Louis, MO, USA). The RNA isolation kit (DP441) was obtained from Tiangen (Beijing, China). Isopropyl β-d-thiogalactopyranoside (IPTG) was purchased from Coolaber (Beijing, China). The TransScript® One-Step gDNA Removal and cDNA Synthesis SuperMix (AT311) was obtained from TransGen Biotech (Shanghai, China).

### 2.2 Sequence alignment and phylogenetic analysis

The sequences of GAOs in Asteraceae were obtained from the National Center for Biotechnology Information (NCBI). Sequence alignment was performed using Jalview software. The phylogenetic tree was constructed using the maximum likelihood method, implemented in the MEGA X program. Amino acid alignment was conducted using ClustalW. Bootstrap values, representing the statistical support for the tree topology, were expressed as percentages based on 1,000 replicates.

### 2.3 RNA extraction and cDNA synthesis

The total RNAs of *A. annua* and *Saussurea costus* were extracted from the leaves using an RNA isolation kit. A total of 1.5 μg of the total RNAs was treated with DNase I (1 U/μl; Fermentas, Waltham, MA, USA) and used for complementary DNA (cDNA) synthesis with oligo(dT) primer using a TransScript® One-Step gDNA Removal and cDNA Synthesis SuperMix.

### 2.4 Protein expression

The open reading frame (ORF) sequences for ADS and GAS were PCR amplified from the cDNAs of *A. annua* as templates. The primers used for amplification can be found in **Supplementary Table 1**. Subsequently, the PCR products were ligated into the *Bam*HI and *Sac*I sites of the pET-32a vector utilizing a homologous recombination system (Paisiwen, Shanghai, China). After successful recombination, the resulting recombinant plasmids were transformed into *Escherichia coli* strain BL21 (DE3) cells for expression. The transformed cells were then grown on Luria–Bertani (LB) medium plates containing 100 μg/mL ampicillin. Four single colonies were selected and inoculated in 3 mL of LB medium containing 100 μg/mL ampicillin. The cultures were incubated for approximately 14 h at 37°C, followed by growth at 1:100 dilution to 600-nm optical density (OD_600_) of 0.8–1.0. Induction of protein expression was achieved by adding 0.25 mM IPTG at 16°C. The recombinant proteins were subsequently purified with Ni-NTA resin (Qiagen, Hilden, Germany) and their concentrations determined using the Bradford method with bovine serum albumin (BSA) as the standard. To verify protein expression, sodium dodecyl sulfate–polyacrylamide gel electrophoresis (SDS-PAGE) was performed using 12% Tris–Gly Protein Gels with 3-(*N*-morpholino)propanesulfonic acid (MOPS) running buffer. The resulting gel was stained with Coomassie Brilliant Blue, and the protein bands were visualized. **Supplementary Figure S1** displays the Coomassie Brilliant Blue staining results of the purified proteins.The ORF sequences for AMOHAP, AMOLAP, and LsGAO (i.e., the GAO in *Lactuca sativa*) were synthesized and cloned into the *Bam*HI and *Eco*RI sites of YeDP60 by Qingke (Beijing, China). The ORF sequence for ScGAO (i.e., the GAO in *S. costus*) was amplified by PCR from the cDNAs of *S. costus* using the primers listed in **Supplementary Table S1**. The PCR products were inserted into the *Bam*HI and *Eco*RI sites of the YeDP60 vector using a homologous recombination system. The recombinant YeDP60s were expressed in *Saccharomyces cerevisiae* WAT11 cells that harbored the *Arabidopsis thaliana* P450 reductase gene, *ATR1*. Yeast growth, induction, and purification of the microsomal proteins were performed according to previous methods (Pompon et al., 1996; Luo et al., 2001).

### 2.5 Enzymatic assay

A reaction system that contains sesquiterpene synthase and cytochrome P450 monooxygenase was used. The reaction buffer (500 μL) contained 25 mM HEPES (pH 7.0), 5 mM magnesium chloride, and 5 mM dithiothreitol. In addition, the reaction mixture included the purified recombinant sesquiterpene enzyme (10 μg) and 10 mg of the crude extract of microsome proteins. To initiate the reaction, 5 μL FPP (1 mg/mL) and 5 μL NADPH (100 mM) were sequentially added. The reaction was then incubated at 30°C for 3 h and layered with hexane (500 μL). As controls, microsomes from yeast containing empty vectors were used. Following the reaction, the mixture was quenched by adding hexane and vortexed for 10 s. Subsequently, it was centrifuged at 12,000 × *g* for 10 min at 4°C. The hexane layer was subjected to GC-MS analysis.

### 2.6 Gas chromatography–mass spectrometry analysis

For GC-MS analysis, we utilized an Agilent 6890 Series GC System with an Agilent 5973 Network Mass Selective Detector and an Agilent HP-5MS column. The HP-5MS column had a composition of 5% phenyl methyl siloxane and dimensions of 30.0 m length, 250.00 μm diameter, and 0.25 μm film thickness. To ensure accurate analysis, we employed a splitless injection technique and utilized helium gas as the carrier gas at a flow rate of 1 mL/min. The enzymatic products underwent analysis using the following temperature program: an initial temperature of 60°C with a 5-min hold, followed by a gradual increase to 270°C at a rate of 10°C/min, and, finally, a rapid ramp to 300°C at a rate of 50°C/min with a 5-min hold.

## 3 Results

### 3.1 Expression patterns of *AMOHAP*, *AMOLAP*, *GAS*, and other artemisinin biosynthesis genes

**Figure 2:**
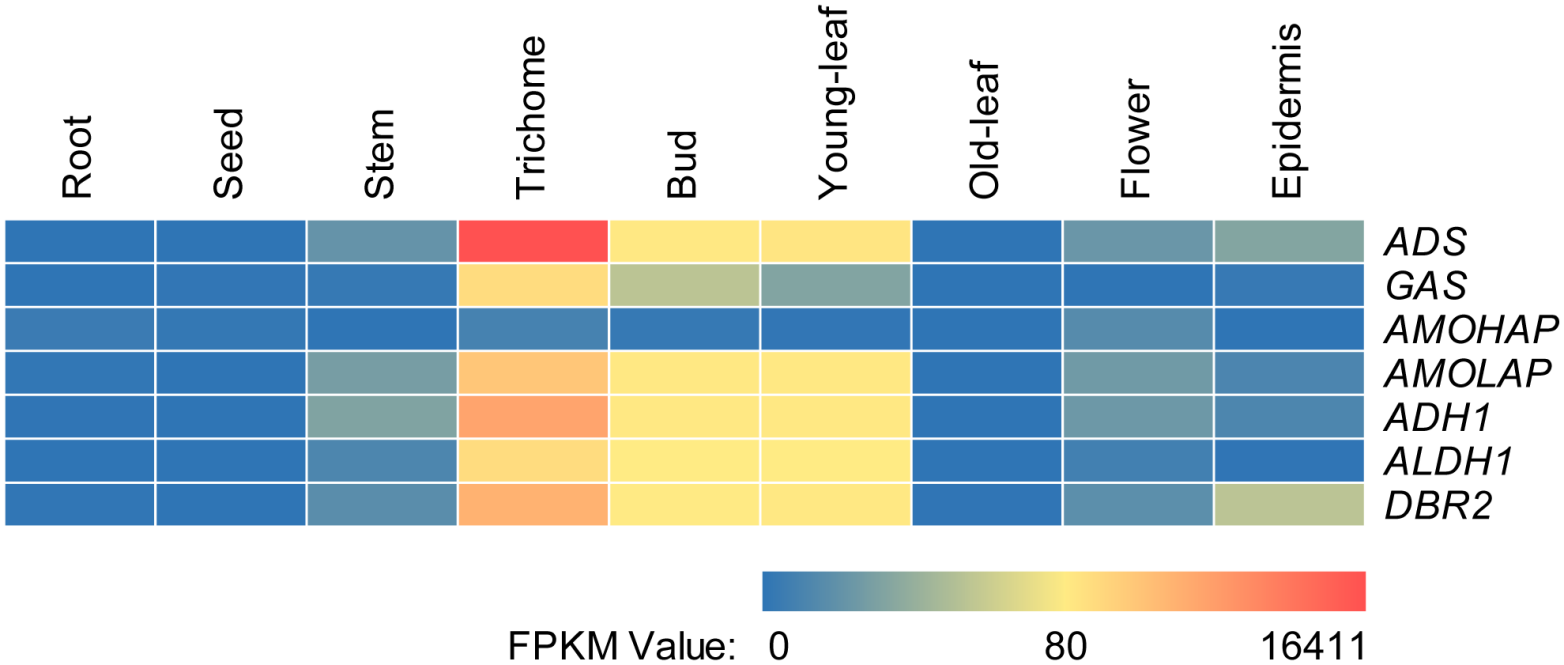
Relative tissue-specific expression levels of the artemisinin biosynthetic genes.

The relative tissue-specific expression levels of the artemisinin biosynthetic genes, including *ADS* (GenBank: PWA56512.1), *AMOHAP* (GenBank: PWA47004.1), *AMOLAP* (GenBank: PWA40082.1), *ADH1* (GenBank: A0A2U1Q018.1), *ALDH1* (GenBank: PWA96689.1), *DBR2* (GenBank: PWA95605.1), and the ASTL gene *GAS* (GenBank: PWA48097.1) were investigated through the analysis of available transcriptomic sequencing data (Shen et al., 2018). As depicted in **Figure 2**, except *AMOHAP*, all artemisinin biosynthesis genes exhibited predominant expression in the trichome, bud, and young leaf, a pattern also observed for *GAS*. However, the transcript of *AMOHAP* was found to be low in all tissues.

### 3.2 Sequence analysis of AMOHAP and AMOLAP

**Figure 3:**
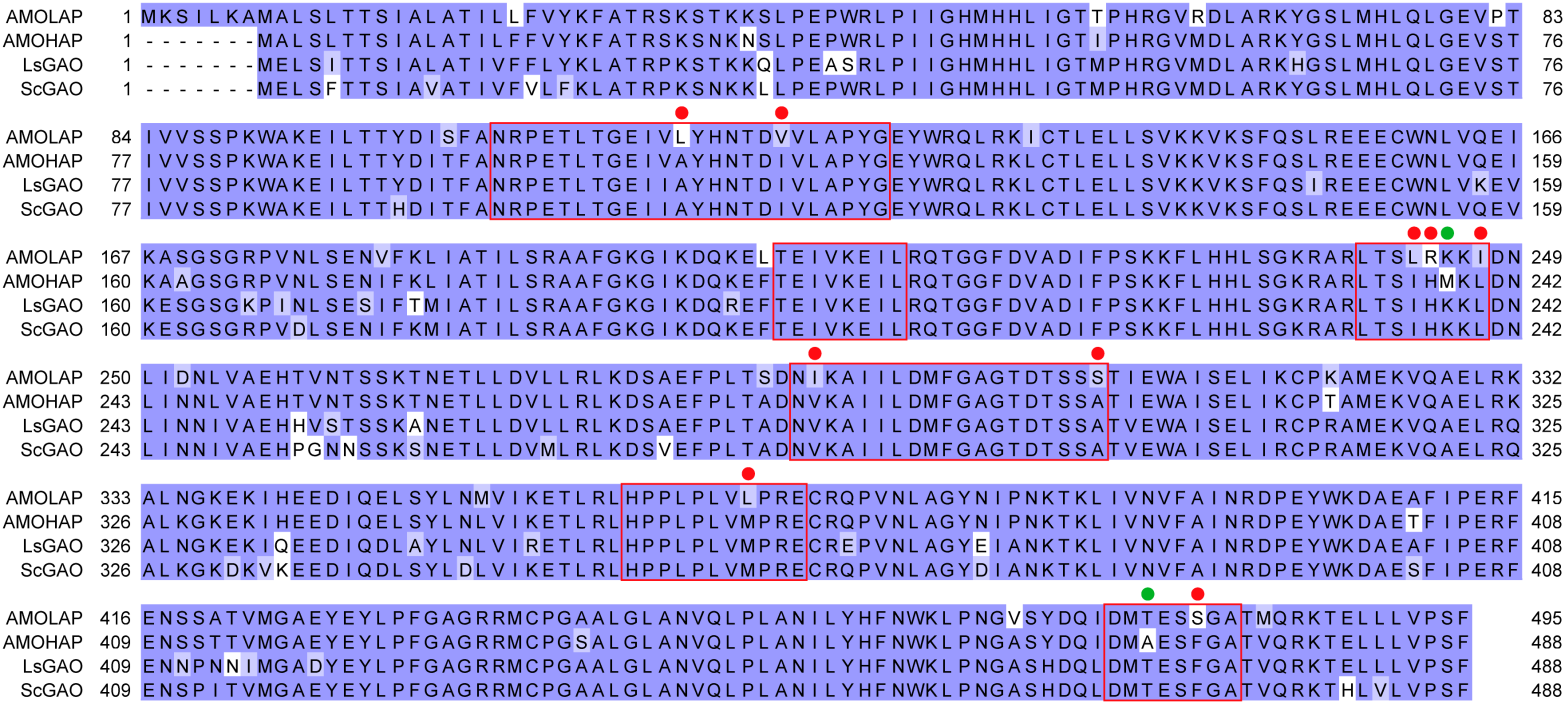
Alignment of the deduced amino acid sequences of AMOLAP, AMOHAP, LsGAO (the germacrene A oxidase in *Lactuca sativa*), and ScGAO (the germacrene A oxidase in *Saussurea costus*). The sequences were obtained from NCBI: LsGAO (ADF32078.1) and ScGAO (ADF43081.1). *Red boxes* denote the predicted catalytic pocket, *red circles* mark the residues conserved in AMOHAP and GAO, but different from AMOLAP, and *green circles* indicate the residues conserved in AMOLAP and GAO, but different from AMOHAP.

To investigate the phylogenetic relationship of AMOHAP, AMOLAP, and other GAOs, the amino acid sequence of AMOHAP was compared with those of AMOLAP and two GAOs from other Asteraceae species, namely, the GAO in *Lactuca sativa* (LsGAO) and the GAO in *S. costus* (ScGAO). The sequence alignments of these four proteins revealed that AMOHAP shares a higher sequence similarity with GAOs compared to AMOLAP. In the predicted substrate recognition region, there were 11 amino acids that differed between AMOHAP and AMOLAP (Nguyen et al., 2019). Notably, 9 of these 11 amino acids of AMOHAP matched those of LsGAO and ScGAO, while only two amino acids of AMOLAP were identical to LsGAO and ScGAO (**Figure 3**). This suggests that the catalytic pocket of AMOHAP retains more features of GAO.

**Figure 4:**
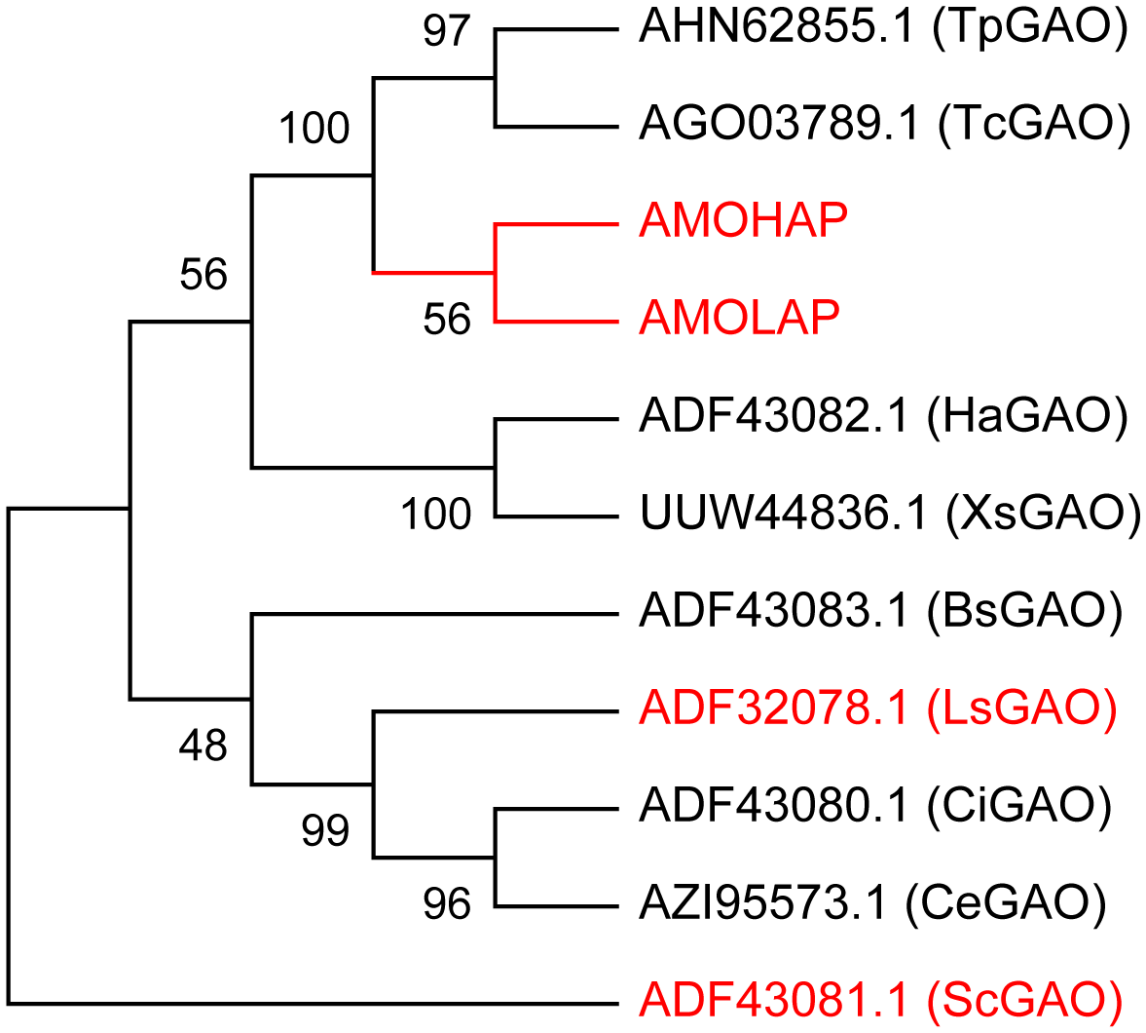
Phylogenetic analysis of AMOHAP, AMOLAP, and the germacrene A oxidases (GAOs) in Asteraceae. The genes used in this study are highlighted in *red*.

We conducted a phylogenetic analysis of GAOs in 10 different plant species, including *Tanacetum parthenium* (Tp), *Tanacetum cinerariifolium* (Tc), *Helianthus annuus* (Ha), *Xanthium strumarium* (Xs), *Lactuca sativa* (Ls), *S. costus* (Sc), *Cichorium intybus* (Ci), *Cichorium endivia* (Ce), and *Barnadesia spinosa* (Bs), comparing them with AMOHAP and AMOLAP proteins. The analysis revealed a close evolutionary relationship between AMOHAP and AMOLAP (**Figure 4**). Since these two proteins are conserved in all ecotypes of *A. annua*, we propose a tentative hypothesis: AMOHAP functions as a GAO, while AMOLAP has evolved new specialized functions derived from GAO.

### 3.3 AMOHAP could catalyze the hydroxylation of germacrene A to germacrene A alcohol

**Figure 5:**
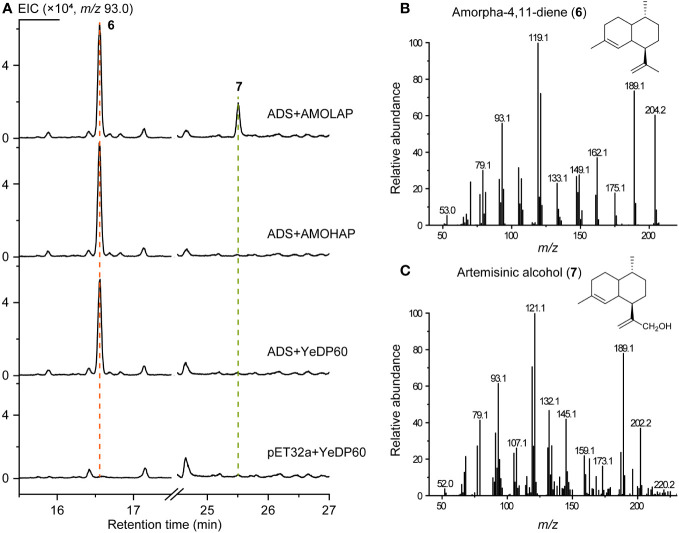
*In vitro* activity of AMOLAP and AMOHAP using amorpha-4,11-diene as the substrate. **(A)** GC-MS traces of the products of recombinant amorpha-4,11-diene synthase (ADS) and AMOLAP- and AMOHAP-containing microsomes. **(B)** Mass spectra of amorpha-4,11-diene. **(C)** Mass spectra of artemisinic alcohol.

To validate the function of AMOHAP, we conducted tests to measure its enzyme activity toward amorpha-4,11-diene and germacrene A. Initially, the ADS expressed by *E. coli* and eukaryotic microsomes expressing AMOHAP and AMOLAP were subjected to an enzymatic reaction using FPP as the substrate. The results revealed the presence of amorpha-4,11-diene (**6**) in both reactions when the AMOLAP and AMOHAP proteins were added. However, artemisinic alcohol (**7**) was only detected when AMOLAP was included (**Figure 5**), confirming the findings of a previous *in vitro* microsome assay (Teoh et al., 2006). These findings suggest that AMOLAP possesses AMO activity, while AMOHAP exhibits negligible activities toward amorpha-4,11-diene under the current reaction condition.

**Figure 6:**
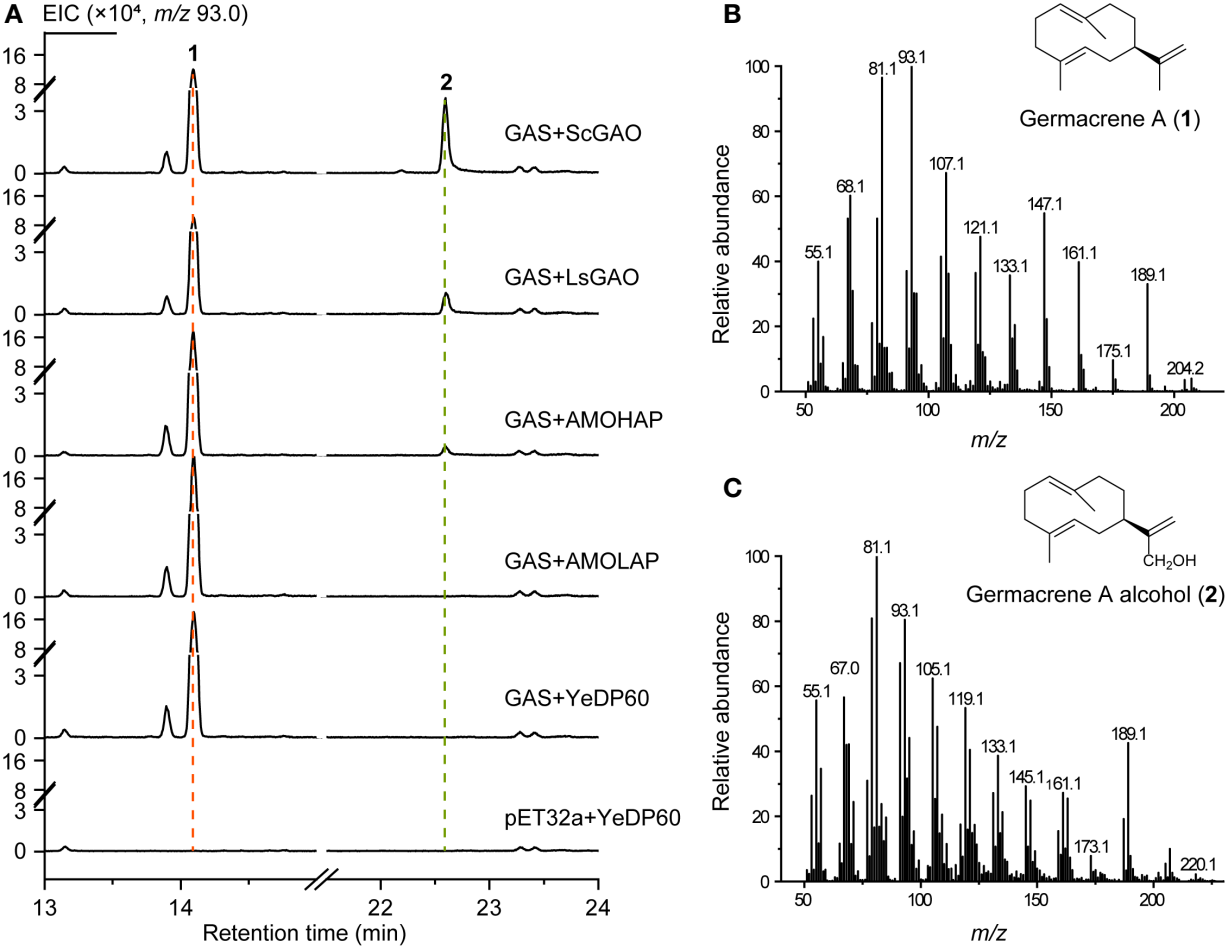
*In vitro* activity of AMOHAP and AMOLAP using germacrene A as the substrate. **(A)** GC-MS traces of the products of recombinant amorpha-4,11-diene synthase (ADS) and AMOLAP- and AMOHAP-containing microsomes. **(B)** Mass spectra of germacrene A. **(C)** Mass spectra of germacrene A alcohol.

Subsequently, the enzyme activity of AMOHAP was examined on germacrene A. The GAS protein expressed by *E. coli* was added to the enzymatic system to convert FPP to germacrene A, which served as a substrate for the microsomes containing AMOHAP and AMOLAP, as well as the ScGAO and LsGAO that were used as positive controls. The results indicated that germacrene A (**1**) and germacrene A alcohol (**2**) were detected in the presence of GAS and the ScGAO- and LsGAO-containing microsomes (**Figure 6**), confirming the effectiveness of the enzymatic assay in catalyzing the conversion of germacrene A to germacrene A alcohol. Similarly, when the AMOHAP-containing microsome was present in the parallel assay, the same result was obtained, while no germacrene A alcohol was detected when the AMOLAP-containing microsomes were added. These results clearly demonstrated that AMOHAP possesses the activity of GAO required for converting germacrene A into germacrene A alcohol.

### 3.4 Promoter variations of AMOHAP and AMOLAP

**Figure 7:**
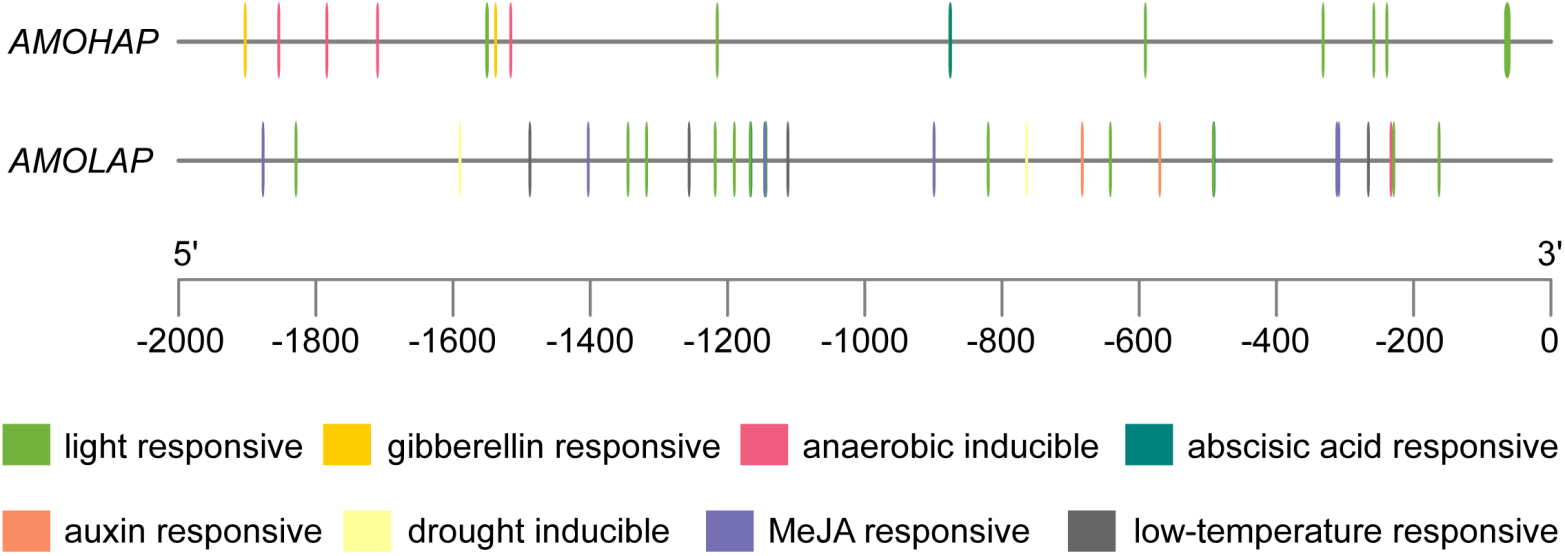
Prediction of the *cis*-acting regulatory elements of *AMOHAP* and *AMOLAP*.

The enzymatic activities of AMOHAP and AMOLAP have been confirmed as GAO and AMO, respectively. However, GAO activity was not detected in *A. annua*, possibly due to the low expression level of *AMOHAP*. To explore the reason for the disparity in expression between *AMOHAP* and *AMOLAP*, we examined the promoter regions of these two genes. Analysis of the *cis*-acting regulatory elements in the promoter regions revealed significant sequence variations, including a greater abundance of light- and abscisic acid-responsive elements in the *AMOLAP* promoter compared to *AMOHAP*. Notably, MeJA- and-auxin responsive elements were exclusively present in the *AMOLAP* promoter, consistent with previous findings that treatment with MeJA can stimulate artemisinin production (**Figure 7**). These differences could have contributed to the distinct expression of these two genes.

## 4 Discussion

Costunolide, the most basic sesquiterpene lactone found in Asteraceae, is widely distributed among various species within this family. The biosynthesis of costunolide starts from FPP and is subsequently catalyzed by GAS and GAO (Nguyen et al., 2010). On the other hand, *A. annua* does not produce costunolide, but contains artemisinin, which is synthesized through the catalytic activities of ADS and AMO (**Figure 1**). It is worth mentioning that GAO exhibits promiscuous activity as it can utilize amorpha-4,11-diene as a substrate to generate artemisinic alcohol, artemisinic aldehyde, and artemisinic acid in yeast. Conversely, AMO shows no activity toward germacrene A, suggesting its functional specialization and loss of ancestral activity (Nguyen et al., 2010; Huang et al., 2021). Previous studies have proposed the existence of two copies of the *CYP71AV1* gene (i.e., *AMOHAP* and *AMOLAP*) in the genome of *A. annua.* Both copies have the ability to produce artemisinic alcohol, artemisinic aldehyde, and artemisinic acid when transiently expressed in *N. benthamiana* (Ting et al., 2013; Shen et al., 2018; Liao et al., 2022). However, the enzymatic activity of AMOHAP on germacrene A has not been analyzed to date. To elucidate the true function of AMOHAP, we conducted an *in vitro* enzymatic assay using both AMOHAP and AMOLAP on germacrene A. Our findings revealed that only AMOHAP, but not AMOLAP, was capable of producing germacrene A alcohol. This result strongly suggests that AMOHAP is indeed a GAO rather than an AMO, and its activity toward amorpha-4,11-diene is a promiscuous activity shared by other GAO enzymes. Therefore, we propose renaming AMOHAP as *A. annua* GAO to accurately reflect its function.

The emergence of a new metabolic pathway can sometimes result in the loss of the ancestral pathway, as is evident in the case of the artemisinin/costunolide pathways in *A. annua*. Germacrene A and functional *GAS* were found to be present in *A. annua*, with the expression of *GAS* being particularly enriched in the trichome, bud, and young leaf, which is consistent with the expression pattern of the artemisinin biosynthetic genes. This observation further supports the idea that costunolide has been replaced by artemisinin in *A. annua.* Therefore, the absence of costunolide biosynthesis in *A. annua* may be attributed to the lack of GAO activity, which is a result of the loss of the *GAO* copy proposed in a previous study (Nguyen et al., 2019). Our findings provide direct evidence that a change in regulation, specifically the reduction in GAO expression, leads to the abolishment of costunolide biosynthesis. Importantly, a natural loss-of-function event is an essential part of the evolutionary landscape; however, previous investigations have primarily focused on the loss of protein-coding genes (Olson, 1999; Sharma et al., 2018; Hecker et al., 2019; Xu and Guo, 2020). Our study highlights that variations in the non-coding upstream region can also result in a reduction of gene expression and loss of function.

Clearly, the real innovation in the emergence of the artemisinin pathway is the advent of ADS activity, which is exclusively found in *A. annua.* Interestingly, the production of amorpha-4,11-diene is a relatively simple innovation. Previous studies conducted by ourselves and others have demonstrated that a single residue switch in α-bisabolol synthase (Fang et al., 2017) and (*E*)-β-farnesene synthase (Salmon et al., 2015) from *A. annua* can generate amorphadiene analogs, such as amorpha-4,7(11)-diene, which only differs from amorpha-4,11-diene in terms of the double bond position. Therefore, the potential ability of sesquiterpene synthase to produce amorphadiene and the promiscuous activity of GAO toward amorphadiene serve as the evolutionary start points for the advent of the artemisinin metabolic pathway in *A. annua*.

## Data availability statement

The original contributions presented in the study are included in the article/**Supplementary Material**. Further inquiries can be directed to the corresponding author.

## Author contributions

XF: Writing – original draft, Writing – review & editing, Conceptualization, Data curation, Funding acquisition, Project administration, Supervision. F-YC: Data curation, Investigation, Writing – original draft, Writing – review & editing. Q-YM: Data curation, Investigation, Writing – review & editing. B-YX: Investigation, Writing – review & editing. Y-CL: Investigation, Writing – review & editing. H-YL: Investigation, Writing – review & editing.
