# Supplementary material for: Functional analysis of CYP71AV1 reveals the evolutionary landscape of artemisinin biosynthesis
Source: Front Plant Sci. 2024 Mar 21;15:1361959. doi: 10.3389/fpls.2024.1361959 (PMC10991709; doi:10.3389/fpls.2024.1361959)
Supplement: Supplementary file 1 [file DataSheet_1.pdf]

Supplementary Material

Functional analysis of CYP71AV1 reveals evolutionary landscape of artemisinin biosynthesis

Fang-Yan Chen, Qiu-Yan Mu, Bing-Yi Xu, Yu-Chen Lei, Hui-Ying Liu, Xin Fang*

*** Correspondence:** Xin Fang: xinfang@mail.kib.ac.cn

Supplementary Figure

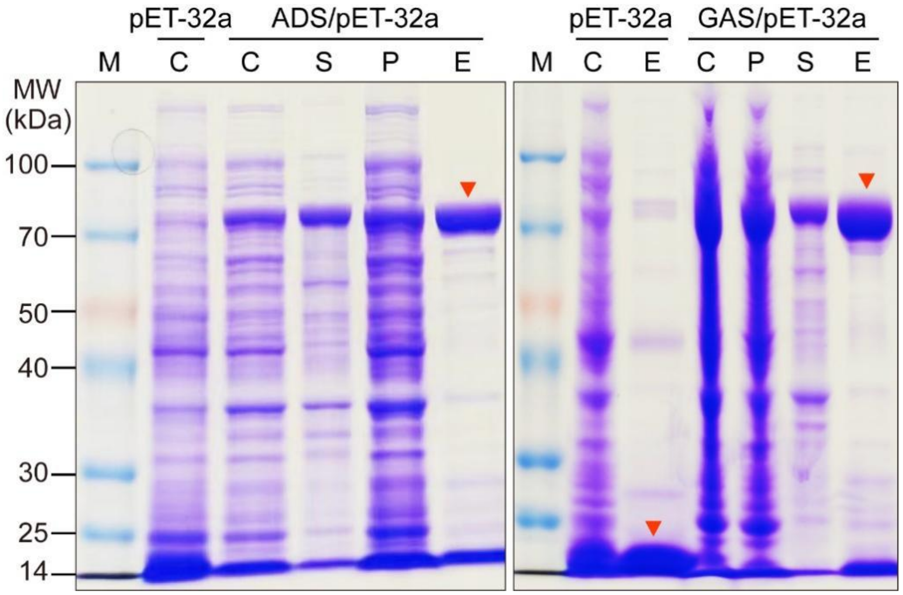

Supplementary Figure 1. SDS-PAGE gels of ADS and GAS proteins. The prokaryotically expressed proteins were purified and subjected to SDS-PAGE gel electrophoresis. The crude (C), supernatant (S), pellet (P), eluate (E) proteins were showed. Red triangles mark the target bands of the indicated proteins.

Supplementary Table 1. Primers used in this investigation.

Gene	Primer Sequence (5'-3')
ADS	F: GCCATGGCTGATATCGGATCCATGTCACTTACAGAAGAAAAACC<br>R: CAAGCTTGTCGACGGAGCTCTCATATACTCATAGGATAAACGAG
GAS	F: GCCATGGCTGATATCGGATCCATGGCAGCGGTTCAAGCTAA<br>R: CAAGCTTGTCGACGGAGCTCTTACACGGGTAGAGAACCCAC
ScGAO	F: ACACACTAAATTACCGGATCCATGGAACTCTCCTTCACCA<br>R: AGATCCCCCGCGAATTCTTAAAAACTAGGTACCAGTACC
